# Real world clinical experience using daily intelligence-assisted online adaptive radiotherapy for head and neck cancer

**DOI:** 10.1186/s13014-024-02436-3

**Published:** 2024-03-30

**Authors:** Philip Blumenfeld, Eduard Arbit, Robert Den, Ayman Salhab, Tal Falick Michaeli, Marc Wygoda, Yair Hillman, Raphael M. Pfeffer, Marcel Fang, Yael Misrati, Noam Weizman, Jon Feldman, Aron Popovtzer

**Affiliations:** 1 Department of Radiation Oncology, Sharett Institute of Oncology, Hadassah Medical Center and Faculty of Medicine, Hebrew University of Jerusalem, POB 12272, 9112002 Jerusalem, Israel; 2 Department of Radiation Oncology, Thomas Jefferson University Hospital, Philadelphia, PA USA

## Abstract

**Background:**

Adaptive radiation therapy (ART) offers a dynamic approach to address structural and spatial changes that occur during radiotherapy (RT) for locally advanced head and neck cancers. The integration of daily ART with Cone-Beam CT (CBCT) imaging presents a solution to enhance the therapeutic ratio by addressing inter-fractional changes.

**Methods:**

We evaluated the initial clinical experience of daily ART for patients with head and neck cancer using an online adaptive platform with intelligence-assisted workflows on daily CBCT. Treatment included auto-contour and structure deformation of Organs at Risk (OARs) and target structures, with adjustments by the treating physician. Two plans were generated: one based on the initial CT simulation with the edited structures (scheduled) and a re-optimized plan (adaptive). Both plans were evaluated and the superior one approved and delivered. Clinical and dosimetric outcomes were reviewed.

**Results:**

Twenty two patients with head and neck cancers (7 Nasopharynx, 6 Oropharynx, 1 oral cavity, 8 larynx) stages I-IVA were treated with daily ART. 770 adaptive and scheduled radiotherapy plans were generated. 703 (91.3%) adaptive plans were chosen. Median time to deliver ART was 20 minutes (range: 18-23). Adaptive compared to scheduled plans demonstrated improved mean V95 values for the PTV70, PTV59.5, and PTV56 by 1.2%, 7.2%, and 6.0% respectively and a mean 1.4% lower maximum dose in PTV70. Fourteen of 17 OARs demonstrated improved dosimetry with adaptation, with select OARs reaching statistical significance. At a median follow up of 14.1 months, local control was 95.5%, two patients developed metastatic disease and four patients died. 9.1% of patients had acute grade 3 dysphagia and 13.6% had grade 2 chronic xerostomia.

**Discussion:**

These findings provide real world evidence of the feasibility and dosimetric benefit of incorporating daily ART on CBCT in the treatment of head and neck cancer. Prospective study is needed to determine if these dosimetric improvements translate into improved outcomes.

## Introduction

Head and neck cancers account for approximately 4% of all cancers globally and are associated with significant morbidity and mortality [1]. Locally advanced head and neck cancers pose a significant challenge due to the complex anatomy and critical structures surrounding the tumor. The management of these cancers often involves a combination of surgery, chemotherapy and radiation therapy. The delivery of accurate radiotherapy is required in order to maximize tumor control while minimizing damage to the nearby structures.

Despite recent technological advancements, locoregional failure occurs in about 30% of patients within 5-years after primary treatment and is a main cause of morbidity and mortality [2, 3]. Additionally, radiation toxicity represents a relevant concern impacting patients’ quality of life even with the use of modern radiation techniques such as intensity modulated radiation therapy (IMRT) and volumetric modulated arc therapy (VMAT). Adaptive radiotherapy (ART), that modifies the radiation delivery during the patient’s treatment course, is emerging as a promising technique to further improve outcomes [4].

ART takes into account the changes occurring during the course of treatment including tumor shrinkage, patient weight loss and anatomic variations in order to improve the precision of the radiation delivery and affect treatment outcomes. Several retrospective studies have demonstrated that ART in head and neck cancers demonstrates improved target coverage and reduction in doses to critical structures [5] and suggest improved local control and reduced toxicity [6]. However, prospective study of weekly adaptive radiotherapy did not demonstrate a benefit in decreasing xerostomia compared with standard IMRT [7].

Given the complexity of replanning head and neck cancer cases, ART is typically only performed once or twice during a patient’s RT course. Recently, the utilization of an intelligence software program to facilitate radiotherapy replanning is a promising solution to improve treatment accuracy and efficiency and potentially improving the therapeutic ratio. A novel online adaptive platform with intelligence-assisted workflows on daily cone-beam computed tomography (CBCT) has become available and has demonstrated feasibility [8, 9].

In this study, we retrospectively reviewed our patients with locally advanced head and neck cancers who were treated with radiotherapy using daily CBCT-based online ART. We assessed the feasibility of this solution by comparing the planned and delivered dose distributions and analyzing the time required to treat patients on this platform. Furthermore, we evaluated treatment oncologic outcomes and treatment-related toxicity.

## Methods

Prior to computed tomography (CT) simulation, all patients are presented at our institutional radiotherapy rounds. Head and neck cancer patients deemed to benefit from daily ART underwent CT-simulation using a 5-point thermoplastic mask for immobilization. CT was performed with and without intravenous contrast (with metal artifact reduction software). Contouring of targets and organs at risk (OARS) was performed on the contrast enhanced CT scan and transferred to the non-contrast CT scan for treatment planning. Gross tumor volume of the primary (GTVp) was defined as the gross extent of the tumor and Gross tumor volume of the nodes (GTVn) defined as all involved regional lymph nodes. Intermediate-risk (IR) and low-risk (LR) clinical target volume (CTV) covering potential microscopic spread, including elective regional lymph node regions were contoured. Planning target volumes (PTV), which include PTV70, PTV59.5 and PTV56, were generated by 3 mm outer margin of GTVp and GTVn, IR-CTV and LR-CTV, respectively and cropped 3 mm from the surface of the body. OARs were delineated by the treating physician and physicist. Specific OARs were chosen as anatomical influencers per head and neck subsite recommendations. A reference 9 or 12 beam IMRT plan was created prescribing 70 Gy, 59.5 Gy and 56 Gy in 35 fractions to respective PTVs and approved by the treating physician using institutional dose/fractionation schema, target goals and OAR constraints. All cases and plans were reviewed by clinicians who would be available for adaptive treatments.

**Fig. 1:**
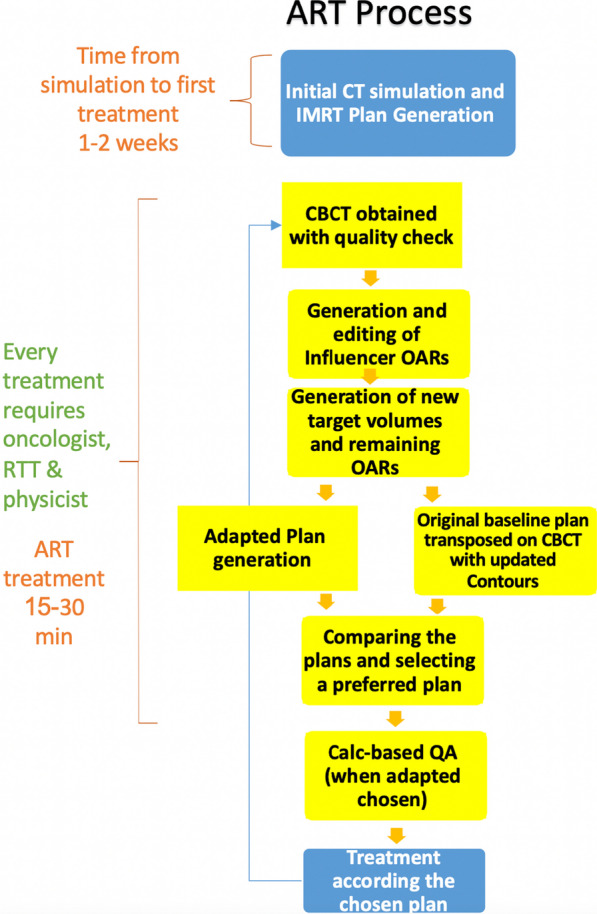
Adaptive process

On the treatment table the patient was aligned and CBCT acquired with optimization of CBCT scanning protocol. The simulation CT scan was registered to the CBCT using deformable registration and a synthetic CT scan was generated. Intelligence-assisted based auto-contour of influencer anatomical structures were checked and edited by the treating physician. Subsequent structure deformation of OARs and target volumes on CBCT were also reviewed and edited by the treating physician. The original GTVp and GTVn volume were preserved utilizing fused pre-treatment positron emission tomography and computed tomography (PET-CT) and Magnetic Resonance Imaging (MRI) images to the CBCT scan when available. If clear gross anatomy differences developed (i.e. air cavities in areas of initial gross disease) the contours were corrected according to daily CBCT. Two plans were generated including, a CT simulation-based plan with deformed structures (scheduled) and a re-optimized plan (adaptive) based on original IMRT planning parameters of which both plans evaluated and the better one approved. If an adaptive plan was chosen, a calculation-based QA was performed and radiation was delivered. CBCT prior to treatment following adaptation was recommended but not mandatory. Figure 1 demonstrates the adaptive process.

Treatment was with chemoradiotherapy or radiation alone depending on the stage and performance status of the patient. Systemic treatment included weekly cisplatin or carboplatin-paclitaxel. Patients were seen weekly during their treatment course and toxicity was recorded by both physician and dietician. Follow-up was performed 1 month post-treatment and every 3 months thereafter. Fiberoptic laryngoscopy was performed and re-evaluation with PET-CT every 3 months.

Dosimetric data collection was performed in order to compare the planned and delivered dose distributions. Target coverage and OAR protection was evaluated for all available contours. Maximum dose to a structure was defined as the dose to the 0.01 cc. Data were analyzed using R statistical software (version 4.2.1). Descriptive statistics were employed to summarize the characteristics of the study population. To account for the correlated nature of the repeated measurements on the same subjects across multiple sessions, linear mixed-effects models were employed. These models were fitted using the lme4 package in R. The significance of the fixed effects was assessed using Wald tests. Model assumptions, including linearity, independence, and normality, were checked through residual plots and other diagnostic tools.

## Results

A total of 22 patients, median age 50 (range 19–81), with locally advanced head and neck cancers (7 Nasopharynx, 6 p16 + Oropharynx, 1 oral cavity, 8 larynx) were treated with daily ART from December 2020 until November 2022. See Table 1 for patient demographics and tumor characteristics. 36.4% of patients had Stage III disease and 31.8% of patients had stage IVA disease. All patients except for one received concurrent chemotherapy with their radiation.

**Table 1:** Patient and treatment characteristics

Characteristic	N (%)
*Sex*	
Male	16 (72.7%)
Female	6 (27.3%)
Age	Median 50 (range 19–81)
*Primary site*	
Oral cavity	1 (4.5%)
Oropharynx	6 (27.3%)
Nasopharynx	7 (31.8%)
Larynx	8 (36.4%)
*T stage*	
T1	3 (13.6%)
T2	6 (27.2%)
T3	11 (50.0%)
T4	2 (9.1%)
*N stage*	
N0	9 (40.9%)
N1	3 (13.6%)
N2	8 (36.3%)
N3	2 (9.1%)
*Group stage*	
Stage I	2 (9.1%)
Stage II	5 (22.7%)
Stage III	8 (36.4%)
Stage IV	7 (31.8%)
*Treatment*	
Chemoradiotherapy	21 (95.5%)
Radiation alone	1 (4.5%)

A total of 770 adaptive and scheduled radiotherapy plans were generated. For 703 (95.7%) fractions the adaptive plan was chosen by the treating physician and physicists. Average time of an adaptive treatment session was 20 min (range 18–23 min). Table 2 includes a dosimetric comparison of adaptive versus scheduled plans as well as the change by session. Adaptive plans demonstrated improved mean V95 for the PTV70, PTV59.5, PTV56 by 1.2%, 7.2%, 6.0% respectively. In the mixed-effects model examining the change in maximum dose of PTV70Gy over treatment sessions, both the treatment group and the session number were significant predictors. Specifically, the adaptive group on average was associated with a 1.4% lower maximum dose of PTV70Gy compared to the non-adaptive group. In addition, for each additional session in the adaptive group, PTV70Gy maximum dose increased by approximately 0.02%. However, the interaction between session and group was not significant, indicating that the rate of change in maximum dose of PTV70Gy over sessions did not differ between the two groups.

**Table 2:** Adaptive versus scheduled (non-adapted) PTV dosimetric comparison

Target	Sessions evaluated	Daily mean difference adapted versus scheduled (CI)	*p*-value	Change per additional session	*p*-value	Interaction between session and group (*p*-value)
Mean PTV70 V95	764	1.2% (0.7–1.6%)	< 0.001	0.003% (− 0.012% to 0.002%)	0.678	0.982
PTV70 Dmax	764	− 1.4% (0.9–1.7%)	< 0.001	0.019% (0.005% to 0.033%)	0.009	0.814
Mean PTV59.5 V95	764	7.2% (5.0–9.5%)	< 0.001	0.2% (− 0.306% to 0.101%)	0.832	0.752
Mean PTV56 V95	662	6.0% (3.0–9.0%)	< 0.001	0.06% (0.03% to 0.09%)	< 0.001	0.261

**Fig. 2:**
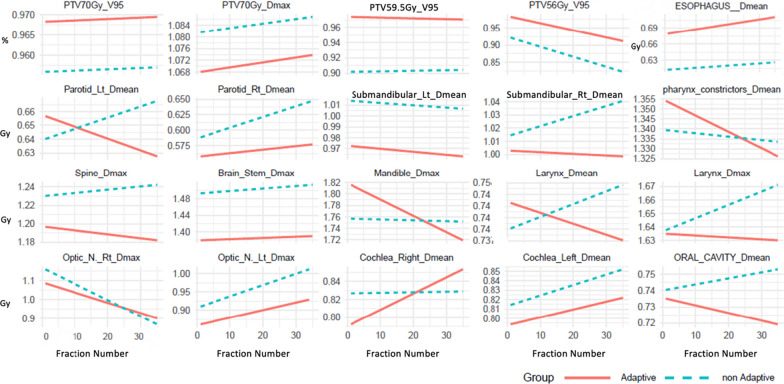
Average daily difference in adaptive versus scheduled for targets (%) and OARs (Gy) over entire treatment course (35 fractions)

Table 3 shows the dosimetric change of select OARs with scheduled versus adaptive treatment including the mean total difference over an entire course (difference × 35 fractions) as well as whether there was a change for each additional fraction delivered. Adaptive treatment resulted in a reduction in maximum dose for spine and brainstem by 1.2 Gy, 3.9 Gy, and an increase in the mandible maximum dose by 2.1 Gy. The left submandibular gland and right cochlea mean dose was reduced by 1.4 Gy and 1.4 Gy respectively and the mean esophageal dose increased by 2.1 Gy. Otherwise all other OARs except for pharyngeal constrictors showed numerical benefit with adaptation, but were not statistically significant. Several OARs demonstrated a statistically significant change per additional session including the right parotid mean, mandible maximum dose, oral cavity mean, pharyngeal constrictor mean, optic nerve mean (right and left), right cochlea mean. Figure 2 graphs the average daily difference (%) in adaptive versus scheduled for PTV70, PTV59.5, PTV56 coverage and maximum dose as well as select OARs over the entire treatment course.

**Table 3:** Adaptive versus scheduled target organ at risk comparison over entire course

Organ at risk	Sessions evaluated	Mean total dose difference (Gy) for adapted versus scheduled (CI)	*p*-value	Change per additional session (*p*-value)	Interaction between session and group (*p*-value)
Left Parotid Mean	750	1.40 Gy (− 6.65 Gy to 3.85 Gy)	0.632	0.753	0.666
Right Parotid Mean	750	0.35 Gy (− 1.05 Gy to 0.18 Gy)	0.148	0.040	0.009
Spine dmax	453	1.16 Gy (0.49 Gy to 1.75 Gy)	0.001	0.231	0.095
Brainstem dmax	347	3.85 Gy (2.80 Gy to 4.90 Gy)	< 0.001	0.761	0.695
Mandible dmax	662	− 2.10 Gy (− 3.50 Gy to − 0.70 Gy)	0.001	< 0.001	0.004
Oral Cavity Mean	695	0.11 Gy (− 0.35 Gy to 0.70 Gy)	0.602	0.022	0.004
Esophagus Mean	585	− 2.10 Gy (− 3.15 Gy Gy to − 1.40 Gy)	< 0.001	0.297	0.360
Left Submandibular Mean	524	1.40 Gy (0.7 Gy to 2.10 Gy)	< 0.001	0.469	0.882
Right Submandibular mean	454	0.35 Gy (− 0.02 Gy to 0.70 Gy)	0.175	0.175	**0.021**
Pharyngeal Constrictors mean	647	− 0.04 Gy (− 1.05 Gy to 0.18 Gy)	0.187	0.002	0.083
Optic Nerve Right Dmax	137	2.45 Gy (− 1.40 Gy to 6.30 Gy)	0.178	0.025	0.266
Optic Nerve Left Dmax	67	1.75 Gy (− 2.10 Gy to 5.25 Gy)	0.389	0.025	0.721
Cochlea Right Mean	312	1.40 Gy (0.07 Gy to 2.45 Gy)	0.035	0.008	0.039
Cochlea Left Mean	277	0.70 Gy (− 0.70 Gy to 1.93 Gy)	0.240	0.404	0.751
Larynx Mean	488	0.21 Gy (− 0.70 Gy to 0.35 Gy)	0.449	0.744	0.184
Larynx Dmax	210	0.05 Gy (− 0.70 Gy to 0.70 Gy)	0.896	0.726	0.048

**Fig. 3:**
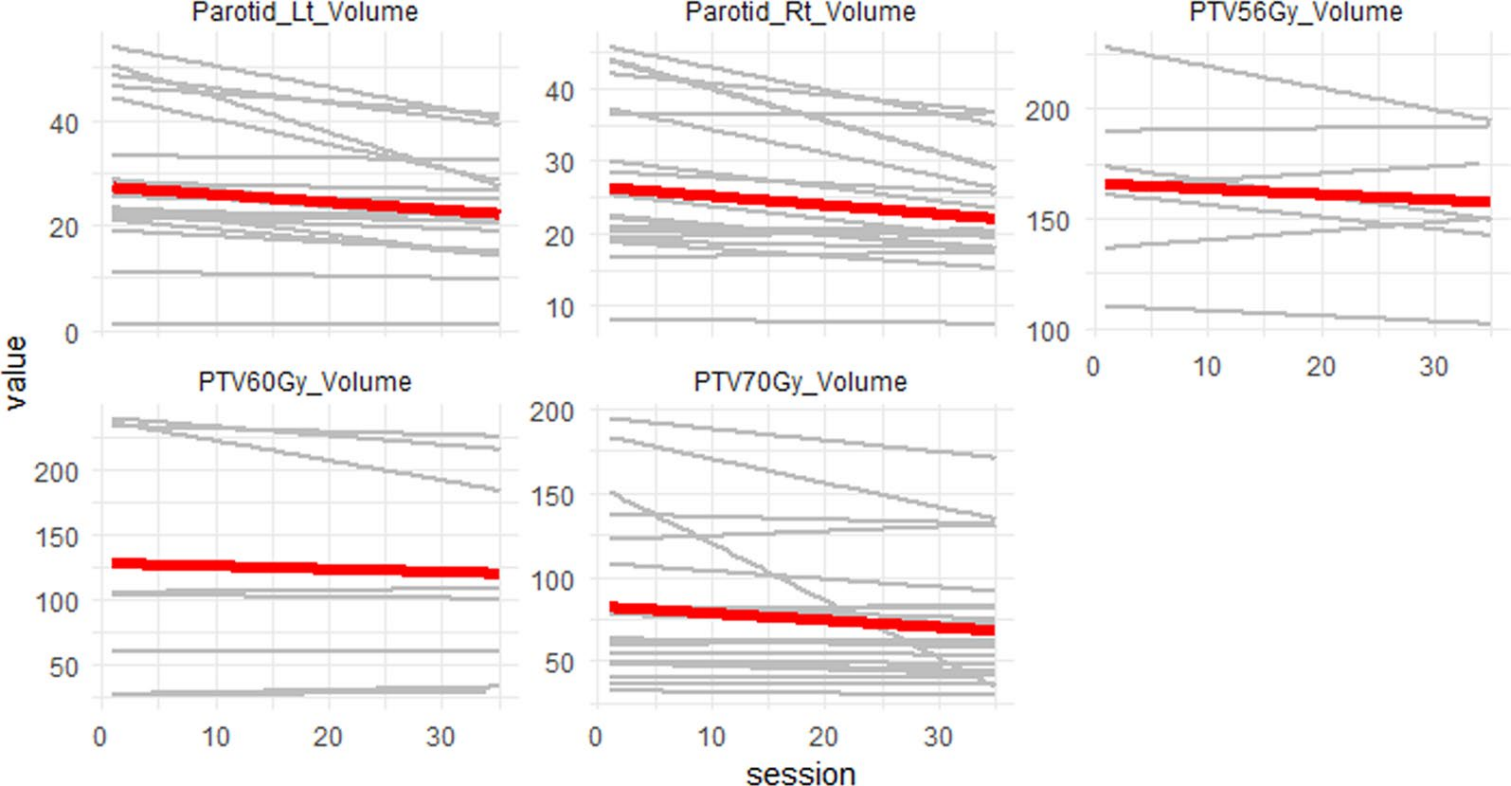
Change in parotid volumes (cc) and PTVs (cc) over treatment course (35 fractions)

Figure 3 shows a plot of the change in parotid volumes (cc) and PTV volumes (cc) over the entire treatment course. There was a statistically significant daily volume reduction for PTV59.5, PTV56, Left Parotid and Right Parotid by 0.28 cc, 0.25 cc, 0.15 cc, and 0.12 cc respectively. PTV70 also demonstrated a reduction in PTV volume but this was not statistically significant.

### Treatment outcomes

At a median follow up of 14.1 months, the local control was 95.5%. 2 patients developed metastatic disease and four patients died (one without evidence of disease at time of death). The single patient with local recurrence had persistent disease following radiotherapy (directly in- high-dose field) and underwent salvage laryngectomy. Three patients required g-tube placement, two prophylactic and one following completion of treatment. Average percent weight loss from start to finish was 5.1% (range 0–11%). 9.1% of patients experienced acute grade 3 dysphagia. Mean percent weight loss 5.1% (range 0–11%). 13.6% with chronic grade 2 xerostomia. There were no grade 4 or grade 5 toxicities. See Table 4 for toxicity outcomes.

**Table 4:** Provider assessed toxicity (CTCAE V.5)

	Any grade toxicity	Grade 1	Grade 2	Grade 3
Skin dermatitis	86.3% (19)	50.0% (11)	31.9% (7)	4.5% (1)
Mucositis	90.1% (20)	45.5% (10)	50.0% (11)	4.5% (1)
Dysgeusia	90.1% (20)	45.5% (10)	40.1% (9)	4.5% (1)
Dysphagia	100% (22)	22.7% (5)	72.7% (16)	9.1% (2)
Acute xerostomia	63.6% (14)	54.5% (12)	9.1% (2)	0
Xerostomia > 3 m	36.3% (8)	22.7% (5)	13.6% (3)	0
Dysphagia > 3 m	27.3% (6)	13.6% (3)	9.1% (2)	4.5% (1)
Fibrosis > 6 m	9.1% (2)	9.1% (2)	0	0

## Discussion

To our knowledge, this is the largest real world study investigating the clinical experience using daily intelligence-assisted online adaptive radiotherapy on cone-beam CT for head and neck cancer available to date including 770 adaptive sessions. We found that daily ART on CBCT improved target coverage for high, intermediate and low-dose PTVs and reduced hot spots in the high dose PTV. In addition, selected OARs were spared with adaptation, although for some OARs this only becomes evident as the patient progresses with treatment (see Fig. 2). Our study cohort predominantly consisted of patients with locally advanced head and neck cancer. Despite the complexity of their cases, we observed excellent local control with only one local failure. Furthermore, our patients experienced favorable toxicity profiles, with few grade 3 toxicities and no occurrences of grade 4 or 5 toxicities. While we have a short median follow up, our initial outcomes appear to compare favorably to other series in terms of local control rates and acute and initial chronic toxicities [10–12].

Adaptive radiotherapy can be based either on functionally-based response using biological imaging such as PET or on anatomical imaging such as magnetic resonance imaging (MRI) or CT [13]. Functional-response based radiation holds promise to improve the therapeutic index through the ability to change dose based on functional imaging or based on response (either via dose escalation or de-escalation) and is currently being evaluated in several prospective studies. Recently published Phase 2 dose escalation study to hypoxic subvolumes using dynamic [18F] fluoromisonidazole PET-CT demonstrated the prognostic ability of such functional imaging whereby dose escalation resulted in a non-significant improvement in local control of 25% [14]. Currently several MRI adaptive studies are underway [15] using both functional and anatomic data. However, such strategies are currently only being implemented with intra-treatment imaging done on a weekly or sporadic basis. Prior to the advent of the CBCT adaptive platform performed in our study, anatomic-based adaptation based on spatial changes throughout the treatment course has been performed on ad-hoc basis or on systematic basis. Retrospective studies of ad-hoc adaptive radiotherapy have demonstrated dosimetric and potential oncologic benefits [5, 6]. Recently, a prospective Phase 3 study on weekly systematic replanning failed to demonstrate improved clinical outcomes compared to non-adapted radiotherapy other than mean parotid excretory function [7]. Our study is one of the first clinical experiences utilizing daily adaptive radiotherapy, a method which has not yet been checked prospectively.

CBCT is widely utilized for daily treatment to correct for setup errors between treatment days [15]. Automated methods for clinical re-planning on each CBCT are now clinically available [16] and was utilized in our study. CBCTs were deemed good enough quality to check the OARs and PTVs and re-contour when necessary. In line with other studies, our analysis demonstrated a statistically significant reduction in target volumes PTV59.5 and PTV56, as well as in the parotid glands, over the course of treatment [17, 18]. While a reduction in PTV70 volume was also observed, it did not reach statistical significance. Importantly, our intention was to preserve the initial gross disease and therefore the observed changes in intermediate and low dose PTVs were more likely attributed, in part, to factors such as weight loss. In our study we were only able to detect a slight dosimetric benefit in the maximum doses to the spine and brainstem. This is likely due to the high prioritization of these serial structures in treatment planning which are carried over with online re-optimization during adaptation as well as the fact that we maintained the volume of the high dose PTV. While most OARs demonstrated improved sparing, these did not reach statistical significance. However, several OARs did demonstrate a statistically significant change per additional session which can be visually appreciated in Fig. 2. We found that one of the significant advantages of utilizing online ART on CBCT is its capacity to provide insights into daily changes in target volumes and organ-at-risk (OAR) structures, enabling the clinical team to comprehend cumulative dose variations throughout the treatment course. This is a departure from traditional assumptions based on contouring and plans established at the time of simulation.

The CBCT workflow introduces additional uncertainty to treatment dose calculation that needs to be considered. The CBCT quality on this system is not considered adequate for direct dose calculation. Therefore, a synthetic CT is generated by the system for dose calculation purposes. The synthetic CT is built using a deformable registration of the planning CT to match the anatomy presented in the daily CBCT. The registration software utilizes regularization rules to ensure that the deformations are anatomically reasonable. For example, voxels cannot cross over each other in the deformation. However, this can lead to inaccuracies in the synthetic CT. For example, Hakansson et al. demonstrated that air cavities which developed over the course of treatment due to shrinking tumor volumes are not reflected in the synthetic CT [19]. Their study shows that these errors in the synthetic CT lead to small uncertainties. Future studies are underway at our institution to further evaluate the impact of synthetic CT inaccuracies.

We acknowledge several limitations in our study. This includes its retrospective nature and the single-institutional design. While we treated a diverse group of head and neck cancer patients, the sample size may have limited our ability to detect more substantial dosimetric benefits, particularly within specific patient subsets. Furthermore, the absence of mandatory additional CBCT scans prior to treatment delivery following adaptation means that potential shifts in patient positioning during adaptation may not have been consistently addressed for some of the fractions. In addition, our analysis only evaluated PTV coverage, although CTV coverage may give a better indication of the value of daily adaptive treatment. In the broader context of head and neck cancer treatment, our study underscores the feasibility of daily adaptive radiotherapy to improve target coverage and potentially improve OARs. However, this advantage does come at the cost of increased total treatment time, affecting both patient comfort and overall resource utilization. Moreover, given that such improvements were minimal and not seen in the majority of the OARs, we question whether utilization of daily ART in all curative intent treatments for head and neck cancer patients will translate into improved outcomes.

Further prospective studies and ongoing research are essential to identify the patient cohorts that may derive the most benefit from daily online ART. Ongoing studies are exploring whether daily online ART may allow for reduced PTV margins thereby reducing long-term toxicity and improving quality of life parameters while maintaining local control [20]. In conclusion, our study represents a substantial step demonstrating the feasibility of daily adaptive radiotherapy for head and neck cancer. It demonstrates the potential to improve radiotherapy, offering improved daily target coverage, which may ultimately improve outcomes for patients facing this challenging disease.

## Data Availability

Research data are stored in an institutional repository and will be shared upon request to the corresponding author.

## Abbreviations

IMRT: Intensity modulated radiation therapy
VMAT: Volumetric modulated arc therapy
ART: Adaptive radiotherapy
CBCT: Cone-beam computed tomography
CT: Computed tomography
GTVp: Gross tumor volume of the primary
GTVn: Gross tumor volume of regional lymph nodes
IR: Intermediate-risk
LR: Low-risk
CTV: Clinical target volume
PTV: Planning target volume
OAR: Organ at risk
PET–CT: Positron emission tomography and computed tomography
MRI: Magnetic resonance imaging
