# Correction: Real world clinical experience using daily intelligence-assisted online adaptive radiotherapy for head and neck cancer

**DOI:** 10.1186/s13014-024-02441-6

**Published:** 2024-05-15

**Authors:** Philip Blumenfeld, Eduard Arbit, Robert Den, Ayman Salhab, Tal Falick Michaeli, Marc Wygoda, Yair Hillman, Raphael M. Pfeffer, Marcel Fang, Yael Misrati, Noam Weizman, Jon Feldman, Aron Popovtzer

**Affiliations:** 1 Department of Radiation Oncology, Sharett Institute of Oncology, Hadassah Medical Center, Faculty of Medicine, Hebrew University of Jeruzalem, POB 12272, 9112002 Jeruzalem, Israel; 2 Department of Radiation Oncology, Thomas Jefferson University Hospital, Philadelphia, PA, United States

**Correction: Radiat Oncol 19, 43 (2024)**

10.1186/s13014-024-02436-3

In this article [1] the abstract was omitted due to a typesetting error and should have appeared as below:

**Abstract**

Background

Adaptive radiation therapy (ART) offers a dynamic approach to address structural and spatial changes that occur during radiotherapy (RT) for locally advanced head and neck cancers. The integration of daily ART with Cone-Beam CT (CBCT) imaging presents a solution to enhance the therapeutic ratio by addressing inter-fractional changes.

Methods

We evaluated the initial clinical experience of daily ART for patients with head and neck cancer using an online adaptive platform with intelligence-assisted workflows on daily CBCT. Treatment included auto-contour and structure deformation of Organs at Risk (OARs) and target structures, with adjustments by the treating physician. Two plans were generated: one based on the initial CT simulation with the edited structures (scheduled) and a re-optimized plan (adaptive). Both plans were evaluated and the superior one approved and delivered. Clinical and dosimetric outcomes were reviewed.

Results

Twenty two patients with head and neck cancers (7 Nasopharynx, 6 Oropharynx, 1 oral cavity, 8 larynx) stages I-IVA were treated with daily ART. 770 adaptive and scheduled radiotherapy plans were generated. 703 (91.3%) adaptive plans were chosen. Median time to deliver ART was 20 min (range: 18–23). Adaptive compared to scheduled plans demonstrated improved mean V95 values for the PTV70, PTV59.5, and PTV56 by 1.2%, 7.2%, and 6.0% respectively and a mean 1.4% lower maximum dose in PTV70. Fourteen of 17 OARs demonstrated improved dosimetry with adaptation, with select OARs reaching statistical significance. At a median follow up of 14.1 months, local control was 95.5%, two patients developed metastatic disease and four patients died. 9.1% of patients had acute grade 3 dysphagia and 13.6% had grade 2 chronic xerostomia.

Discussion

These findings provide real world evidence of the feasibility and dosimetric benefit of incorporating daily ART on CBCT in the treatment of head and neck cancer. Prospective study is needed to determine if these dosimetric improvements translate into improved outcomes.

The original article has been updated.
